# Regulation of *corA*, the Magnesium, Nickel, Cobalt Transporter, and Its Role in the Virulence of the Soft Rot Pathogen, *Pectobacterium versatile* Strain Ecc71

**DOI:** 10.3390/microorganisms11071747

**Published:** 2023-07-04

**Authors:** Caleb M. Kersey, C. Korsi Dumenyo

**Affiliations:** 1 Department of Biological, Physical and Human Sciences, Freed-Hardeman University, Henderson, TN 38340, USA; 2 Departments of Plant Science, Tennessee State University, Campus Box 9543, Nashville, TN 37209, USA

**Keywords:** *Pectobacterium*, soft rot, magnesium transport, CorA, HrpL

## Abstract

*Pectobacterium versatile* (formally *P. carotovorum*) causes disease on diverse plant species by synthesizing and secreting copious amount of plant-cell-wall-degrading exoenzymes including pectate lyases, polygalacturonases, cellulases, and proteases. Exoenzyme production and virulence are controlled by many factors of bacterial, host, and environmental origin. The ion channel forming the magnesium, nickel, and cobalt transporter CorA is required for exoenzyme production and full virulence in strain Ecc71. We investigated CorA’s role as a virulence factor and its expression in *P*. *versatile*. Inhibiting the transport function of CorA by growing a CorA^+^ strain in the presence of specific CorA inhibitor, cobalt (III) hexaammine (Co (III)Hex), has no effect on exoenzyme production. Transcription of *pel-1*, encoding a pectate lyase isozyme, is decreased in the absence of CorA, suggesting that CorA influences exoenzyme production at the transcriptional level, although apparently not through its transport function. CorA^−^ and CorA^+^ strains grown in the presence of Co (III)Hex transcriptionally express *corA* at higher levels than CorA^+^ strains in the absence of an inhibitor, suggesting the transport role of *corA* contributes to autorepression. The expression of *corA* is about four-fold lower in HrpL^−^ strains lacking the *hrp*-specific extracytoplasmic sigma factor. The *corA* promoter region contains a sequence with a high similarity to the consensus Hrp box, suggesting that *corA* is part of Hrp regulon. Our data suggest a complex role, possibly requiring the physical presence of the CorA protein in the virulence of the *Pectobacterium versatile* strain Ecc71.

## 1. Introduction

The soft rot Pectobacteriaceae is a group of phytopathogenic bacteria that produce plant-cell-wall-degrading enzymes (PCWDEs), the actions of which lead to cell wall collapse and tissue maceration in a wide number of plant hosts [1,2,3]. Bacteria in this group have been through a series of revisions over the past decade or so at the species, genus, and family levels [4]. Members of this group now belong to 19 species of the genus *Pectobacterium* and 12 species of *Dickeya.* Strain Ecc71, previously classified as *Erwinia carotovora* subspecies carotovora, has now been assigned as *Pectobacterium versatile* [5]. Soft rot Pectobacteriaceae produce many virulence factors including plant-cell-wall-degrading enzymes such as the pectinases pectate lyase (Pel) and polygalacturonase (Peh), cellulases (Cel), and proteases (Prt), which are ultimately responsible for the manifestation of the soft rot disease in host plants, which include many economically important crops [6,7]. In addition to environmental factors, exoenzyme synthesis and virulence in *P. versatile* is controlled by a complex network of positive and negative regulatory genes, and chemical signals of both bacterial and host origin [1,8,9,10]. The number of regulators controlling virulence and exoenzyme production in soft rot bacteria now number in the twenties. Some of the regulatory systems controlling soft rot virulence include the quorum-sensing system, regulator of secondary metabolism (Rsm) system, Gac (GacS and GacA) system, KdgR (2-keto-3-deoxygluconate repressor), and sigma factors such as the Hrp-specific sigma factor HrpL, whose effect is not through PCWDE production but the *hrp/hop* regulon [11,12].

For the bacterium to produce disease in the host, these enzymes are synthesized and secreted outside the cell by type I (Prt), type II (Pel, Peh, and Cel), type III (harpin), and type VI secretion systems, which bring various substrates, including the enzymes, into contact with substrates in the plant tissue [13,14,15]. In addition to the canonical secretion systems, other transport proteins have been reported to be necessary for full virulence in soft rot Pectobacteriaceae and these include transporters of secondary metabolites [16,17,18,19], as well as transporters involved in nutrient uptake [20], multidrug resistance [21,22], and osmoprotection [23].

A *P. versatile* mutant in the magnesium-transporting membrane protein, CorA, is reduced in the production of the major exoenzymes (Pel, Peh, Cel, and Prt), and less virulent in celery and carrot [24]. The production of *pel-1*, *peh-1*, *celV*, and *prtW* transcripts were each dependent on the presence of CorA, as the absence of CorA was accompanied by a decrease in the levels of transcripts of these exoenzyme genes in comparison with that of *recA*, which was not affected. A CorA^−^ mutant carrying a functional *corA^+^* plasmid was restored to exoenzyme production and virulence, establishing CorA’s involvement in pathogenicity. That the CorA mutation impacts all the major exoenzymes suggests a vital role for CorA in *P. versatile*.

Due to the essentiality of magnesium in the cellular metabolism of organisms in all three domains of life, Mg^2+^ is the most abundant divalent cation in the cell and the second most abundant cation after K^+^ [25]. Due to its importance, many Gram-negative bacteria have redundant magnesium transport systems. These are made up of the major transporters, CorA and MgtE, and the P-type ATPases MgtA/MgtB and their associated MgtS. MgtS forms a complex with a cation–phosphate symporter, PitA, to stabilize MgtA [26]. Lastly, there is the bacterial homologues of natural resistance-associated macrophage proteins (Nramp)-related protein previously shown to transport divalent cations of transition metals [5,27,28]. In addition to CorA, the genome of *P. versatile* has homologues of the MgtA/MgtB system and MgtE, which is almost as common in bacteria as CorA [27]. Despite their genetic diversity, MgtE complements growth in a CorA–MgtA–MgtB *Salmonella* strain [29]. This suggests that such redundancy could be functional in magnesium transport in *P. versatile* as well.

The presence of MgtE, another major Mg^2+^ transporter in *P*. *versatile*, adds to the complexity of its magnesium transport system and necessitates investigation into links between the *corA^−^* phenotype of reduced virulence and intracellular Mg^2+^ concentration. Understanding the mechanism(s) by which CorA is involved in virulence in *Pectobacterium* is complicated by the redundancy of magnesium transporters in *P. versatile.* Microarray data of a CorA mutant of *S. enterica* serovar Typhimurium revealed differential expression of genes with roles in host infection, intracellular survival, metabolism, and transcriptional regulation [30]. As exoenenzyme production and virulence in *P. versatile* are largely controlled by a network of many transcriptional regulators, it is possible that CorA’s effect on *P. versatile* virulence may be through some of these known regulators. The aim of this study was to further investigate CorA’s regulation and its role in exoenzyme production and virulence in *P*. *versatile*. 

## 2. Materials and Methods

### 2.1. Bacterial Strains, Plasmids, and Media

Bacterial strains and plasmids used in this study are listed in Table 1. Bacteria carrying drug resistance markers were maintained at 28 °C in minimal medium (MM) or LB medium containing the appropriate antibiotics. When required, chloramphenicol (Cm, 15 µg mL^−1^), tetracycline (Tc, 10 µg mL^−1^), and CoCl_2_ (50.0 μM) were added to the medium. Media were solidified by the addition of 1.5% agar (*w*/*v*). The compositions of LB and MM were described previously [24,31]. Minimal medium was supplemented with increasing MgSO_4_ concentrations of 1× (0.4 mM), 2× (0.8 mM), 3× (1.2 mM), 4× (1.6 mM), and 10× (4.0 mM). A stock solution of Co(III)Hex was prepared by filter sterilizing a 10.0 mM solution through a 0.2 μM filter and adding to MM at final concentrations of 250.0 μM. For liquid cultures, bacteria were grown in MM at 28 °C at 200 rpm and growth was monitored with Klett colorimeter. Minimal-medium-grown culture samples were harvested at 250 Klett units for assays for enzymatic activities.

### 2.2. Enzyme Assays

Pectate lyase (Pel) and protease (Prt) activities were measured quantitatively from culture supernatants of MM-grown cultures as previously described [24]. All β-galactosidase assays were performed from overnight (16 h)-grown LB medium cultures as described by Miller [42]. Developed samples for quantitative Prt assays were placed in a microtiter plate and A_420_ was measured in a Spectra Max M5 microplate reader (Molecular Devices, San Jose, CA, USA). Beta-galactosidase enzymatic activity is expressed in Miller units. All experiments were repeated three times and the data presented represent average of three biological replicates.

### 2.3. Molecular Techniques

All DNA manipulations including genomic DNA isolation, gel electrophoresis, and bacterial transformation by electroporation were performed by standard methods [31]. For plasmid isolation, either pure yield Plasmid Miniprep kit (Promega, Madison, WI, USA) or the alkaline lysis method [31] was used. Biparental matings were performed with *E. coli* S17-1 λPir as the donor strain. All RNA extractions were performed using a modified hot phenol–chloroform extraction method [24,43]. Nucleic acid quantification was performed on a NanoDroP ND-100 spectrophotometer (NanoDroP Technologies, Wilmington, DE, USA). After total RNA was quantified, 20 µg of RNA was DNase-digested using a TURBO DNA-*free*™ kit (ThermoFisher, Waltham, MA, USA). To verify the absence of any detectable genomic DNA, PCR was performed on DNase-digested RNA.

### 2.4. Construction of P. versatile KD103

To generate a *lacZ^−^* and *corA*^−^ double-mutant strain of *P. versatile*, the plasmid pCKD122 containing *corA^+^* DNA in pRK415 was mutagenized using the EZ::TN <KAN-2> insertion kit (Lucigen, Madison, WI, USA). According to manufacturer’s recommendations, 2.0 μg of pCKD122 was mixed with EZ::TN <Kan-2> transposon and EZ::TN transposase in its corresponding buffer, and incubated at 37.0 °C for 2 h. To stop the reaction, 1.0 μL of stop solution was added and incubated at 70 °C for 10 min. Electrocompetent cells of *E. coli* XL-10 Gold were electroporated with 3.0 uL of transposition mix. After an hour incubation at 37.0 °C, cells were plated on LB containing Km. Selected transformants were screened for Km insertion in the *corA* gene by PCR using the primers corA-qRTPCR-P1 (5′ GTCTTCCGGCTCGATCAAAT3′) and EZ::TN ME (5′ AGATGTGTATAAGAGACAG 3′). The plasmid pCKD123 was confirmed to have Km insertion in *corA*, and conjugated into KD100 by biparental mating. KD103 was generated by marker exchange by patching mating transformants on Km and Tc plates, and selecting for a Tc-sensitive, Km-resistant colony. KD103 *corA::EZTN*, Km^R^ genotype and *corA*^−^ phenotype were confirmed.

### 2.5. RT-qPCR

Gene expression as measured by RT-qPCR was performed as previously described using a SYBR Green-based system [24]. The following primers (listed 5′ to 3′) were used in RT-qPCR. corA-qRTPCR-P1: GTCTTCCGGCTCGATCAAAT, corA-qRTPCR-P2: CTGAGCGCATTTAAACTGGA, hrpN-qRTPCR-P1: ATGAGCGTTGGGCAAAAAG, hrpN-qRTPCR-P2: GGATATTGATCCATAAACTGACCA, Pc-RecAP1: GGTGAGCTGGTTGATCTGGG, Pc-RecAP2: GCATTTGCTTTGCCCTGACC. 

### 2.6. Inductively Coupled Plasma Optical Emission Spectrometry (ICP-OES)

Parental strain KD100 and CorA^−^ mutant KD101 were each grown in 15.0 mL of MM and cultures were harvested at 250 Klett units. A 10.0 mL sample of each culture was centrifuged at 8500 rpm for 10 min and the supernatant was discarded. Pelleted cells were washed in TE (10.0 mM Tris-HCl, pH 8.0; 0.1 mM EDTA) and centrifuged at 14,000 rpm for 10 min. The supernatant was removed, and this step was repeated two times. Cells were then dried by speed vacuum centrifugation. A 200 µL sample of 16 M nitric acid was added to the cells and digested at 80 °C for 1 h. Water was added to the digested cells to a final volume of 10.0 mL. Total magnesium, nickel, and cobalt concentrations in the samples were measured by ICP-OES analysis. A standard curve of magnesium, cobalt, and nickel was each generated and used to determine the concentrations of these metals within the cell. Concentrations are expressed as ppm/A_600_.

## 3. Results

### 3.1. Intracellular Mg^2+^ Concentrations in a CorA^−^ Mutant

The *corA^−^* mutant of *P. versatile* is deficient in the production of plant-cell-wall-degrading enzymes and in virulence [24]. Since CorA is a primary Mg^2+^ transporter, we considered the possibility that the reduction in exoenzyme production in the *corA^−^* strain is the result of a deficiency in intracellular Mg^2+^ resulting from lack of Mg^2+^ transport into the cell. Although the alternate Mg^2+^ transporters, MgtA, MgtB, and MgtE, in *P. versatile* should functionally complement the absence of CorA in Mg^2+^ influx, and we have previously established that the growth of a *corA* mutant is no different from its parent [24], we wanted to verify that the CorA^−^ phenotype was not caused by Mg^2+^ deficiency in the cell resulting from lack of Mg^2+^ uptake. To confirm that there was no Mg^2+^ deficiency in the CorA^−^ strain, we grew parent KD100 and its CorA^−^ mutant KD101 in MM containing normal concentration (0.4 mM) of Mg^2+^. We find no difference in total intracellular Mg^2+^ concentration between the *corA^−^* mutant (KD101; 6.70 ± 0.06 ppm/A_600_) and its parent (KD100; 6.73 ± 0.37 ppm/A_600_). These data suggest that the altered phenotype seen in a CorA^−^ mutant of *P. versatile* is not due to lack of Mg^2+^ in the cell. As CorA also functions in the transport of Ni^2+^ and Co^2+^, the intracellular concentrations of these cations were also measured in the CorA^+^ and CorA^−^ strains. However, Ni^2+^ and Co^2+^ were predictably not detected because MM medium contains neither element. Although we cannot rule out the deficiency of Ni^2+^ and Co^2+^ in the CorA^−^ mutant phenotype, we believe it is unlikely their deficiencies are responsible for a CorA^−^ phenotype, as exoenzyme production in this mutant is also reduced in our defined minimal medium, which does not contain these metals.

### 3.2. Effect of CorA Inhibitor on Exoenzyme Production

**Figure 1:**
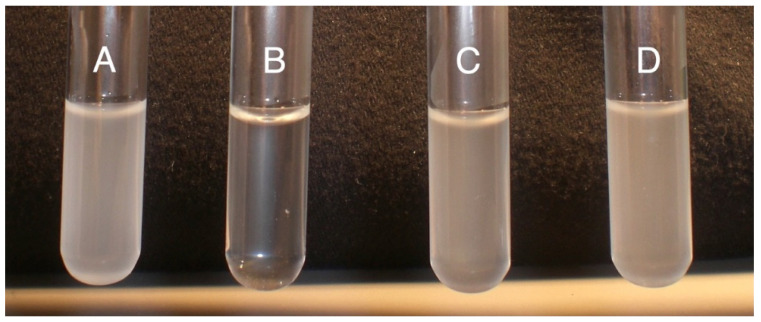
Growth of *P. versatile* strains in the presence of cobalt and CorA inhibitor. (**A**) CorA^−^ strain (KD101/pTH19cr) grown in 50 µM CoCl; (**B**) CorA^+^ (KD101/pCKD121) strain grown in 50 µM CoCl; (**C**) CorA^+^ strain (KD100) grown in 50 µM CoCl + 250 µM Co(III)Hex (CorA inhibitor); (**D**) CorA^−^ (KD101/pTH19cr) strain grown in CoCl + 250 µM Co(III)Hex. The experimental setup is also shown in the table below. This experiment was repeated three times each time with triplicate replicates with similar results. Please see Table 1 for the relevant genotypes of all the bacterial strains used in the study.

**Figure 2:**
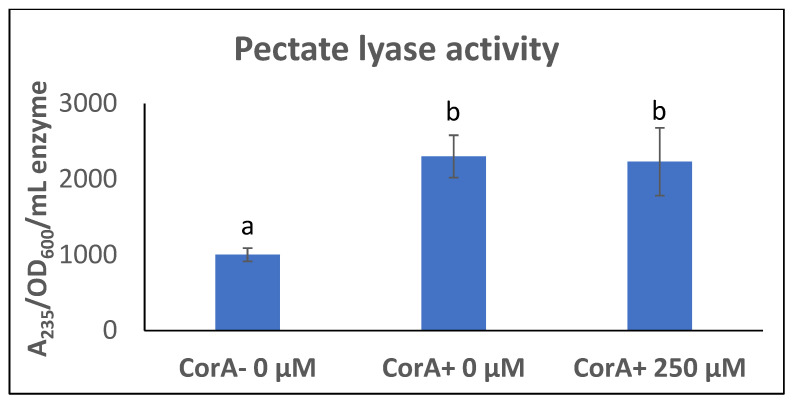
Pectate lyase activity in the presence of CorA inhibitor. CorA^−^ (KD101/pTH19Cr) and CorA^+^ (KD101/pCKD121) strains of *P. versatile* were grown in minimal media supplemented with or without CorA inhibitor Co(III)Hex and Pel activity was determined from culture supernatants. Data represent the means of three biological replicates. Letters that are not the same indicate a significant difference where *p* < 0.05 as determined by a Student’s *t*-test.

CorA^−^ phenotype in *P*. *versatile* is associated with approximately half the amount of exoenzymes as compared to the CorA^+^ parent [24]. As CorA is a transporter, we wanted to determine if the reduced exoenzyme production in CorA^−^ *P*. *versatile* is connected to CorA’s transport function. We reasoned that if CorA’s transport function is influencing exoenzyme production, we could mimic a *corA* mutation by blocking the transport function of CorA in a *corA^+^* background. It has previously been reported that Co(III)Hex inhibits CorA transport system in *S. enterica* serovar Typhimurium [44]. Being structurally analogous to CorA substrates, this chemical presumably binds to and blocks the membrane ion CorA channels. We reasoned that adding Co (III) Hex would mimic a condition where CorA transport is inhibited and, thus, we first wanted to establish that in *P. versatile*, Co (III)Hex similarly inhibits the transport function of CorA. As shown in Figure 1, CorA^+^ and CorA^−^ were grown in MM supplemented with both Co^2+^ and Co(III)Hex. As seen in Figure 1 (tubes A and B), the growth of CorA^−^ in the presence of 50 µM CoCl_2_ is not affected while growth of CorA^+^ is inhibited by 50 µM CoCl_2_. This is expected as cobalt is a toxic metal. However, when 250 µM Co (III)Hex is supplemented into the medium already containing 50 µM CoCl_2_, growth is restored to the CorA^+^ (tube C). We, therefore, conclude that growing *P. versatile* in Co (III)Hex generates a blocked CorA channel, preventing cobalt uptake. Since both our CorA^+^ and CorA^−^ strains possess alternate magnesium transporters, growing a CorA^+^ strain in the presence of the CorA inhibitor does not affect growth (data not shown). However, if the decreased exoenzyme phenotype of *corA^−^* strains is dependent on the transport function of CorA, we predict that exoenzyme production is decreased when wildtype CorA^+^ is grown in the presence of the CorA inhibitor, because the inhibition CorA’s transport function would mimic a *corA* mutation and result in a strain with reduced exoenzyme production. Surprisingly, as seen in Figure 2, in the presence of the inhibitor, pectate lyase activity in a CorA^+^ strain is not different from the activity in a CorA^+^ strain grown without the inhibitor. While the same phenotype of growth in cobalt is seen in a CorA^−^ strain and a CorA^+^ strain grown with Co (III)Hex (Figure 1), the same reduced exoenzyme phenotype of a CorA^−^ mutant could not be mimicked by interfering with the transport function of CorA in a CorA^+^ strain.

### 3.3. CorA Influences Exoenzymes at the Transcriptional Level

**Figure 3:**
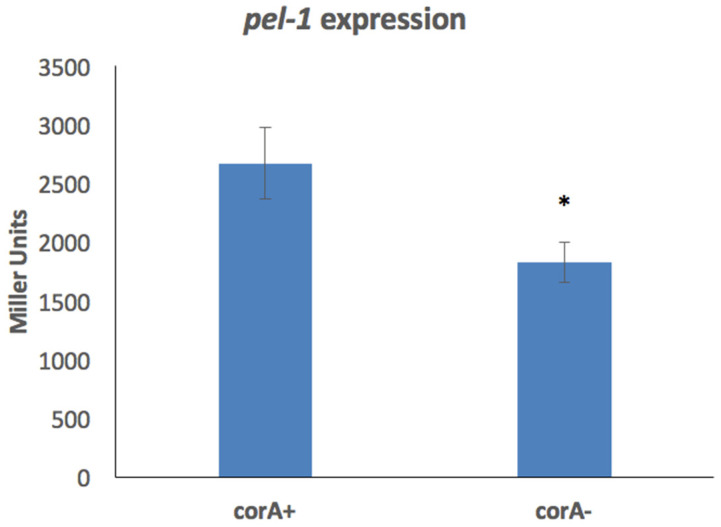
Expression of *pel*-*1* in the absence of *corA*. KD100/pAKC1203 (CorA^+^) and KD103/pAKC1203 (CorA^−^) strains both carrying *pel-1* promoter cloned in lacZ reporter plasmid pMP220 were grown in minimal medium and expression of *pel-1* was measured by Miller assay. Data represent the average of three biological replicates. * indicates a significant difference where *p* < 0.05 as determined by a Student’s *t*-test.

It was previously reported that a *corA* mutant in *P*. *versatile* is reduced in the production of exoenzymes, and established that transcript levels and exoenzyme activity are decreased when CorA is absent [24]. As the production of enzymes can be regulated at many points, we wanted to determine at what level of gene expression CorA affects exoenzyme production. As the first regulatory step is transcription, we investigated the transcription of the *pel-1* gene, which encodes the Pel-1 isozyme of the exoenzyme pectate lyase in parental KD100 (*lacZ^−^*) and mutant KD103 (*lacZ*^−^ *corA*^−^) using the plasmid pAKC1203 carrying *pel-1::lacZ* fusion. As seen in Figure 3, the expression of *pel*-*1* is significantly decreased in the *corA* mutant as compared to its parent. This suggests that CorA’s influence on *pel-1* and possibly the genes of other exoenzyme such as protease, polygalacturonase, and cellulase which are similarly affected by *corA* mutation is at the transcriptional level.

### 3.4. corA Expression in P. versatile

Having established that *corA* influences the transcription of exoenzyme, *pel-1* gene and possibly other exoenzyme genes, we wanted to investigate the expression of *corA* itself. The Tn5 *lacZ*1 transposon used to generate the *corA* mutation in KD101 provided a *lacZ* transcriptional fusion driven by the *corA* promoter [24]. Additionally, the CorA^−^ mutant KD101 complemented with a functional *corA* gene had the production of both exoenzymes, pectate lyase and protease, and pathogenicity restored to wildtype levels. Thus, two systems with a chromosomal *corA-lacZ* transcriptional fusion allowed for *corA* expression studies in both a CorA^−^ and CorA^+^ background.

**Figure 4:**
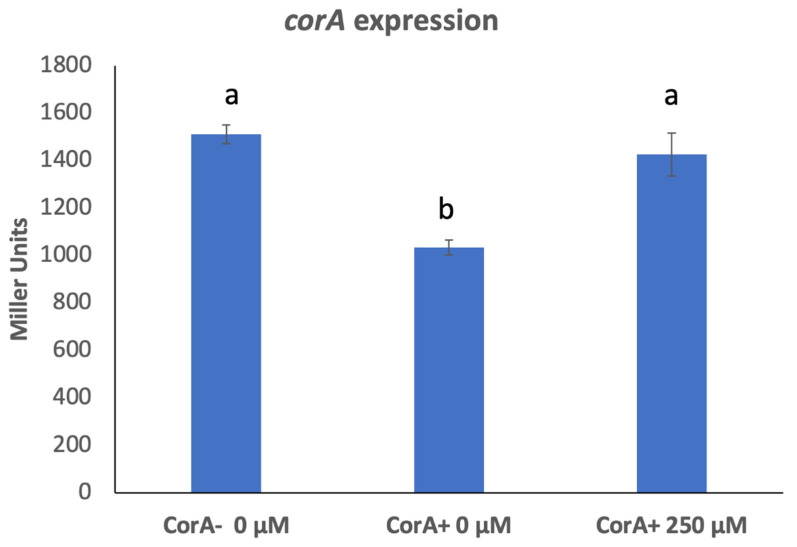
Expression of *corA* in the presence of CorA inhibitor. CorA^−^ KD101with chromosomal *corA-lacZ* fusion carrying either cloning vector, pTH19cr or pCKD121 (*corA^+^*), were grown in media supplemented with a range of Co(III)Hex concentrations and β-galactosidase activity was determined. Data represent the means of three biological replicates. Letters that are not the same indicate a significant difference where *p* < 0.05 as determined by a Student’s *t*-test.

As measured by β-galactosidase activity, there is an increase in the expression of *corA* in a CorA^−^ mutant background (KD101/pTH19cr) compared to its expression in a CorA^+^ background (KD101/pCKD121) (Figure 4). To confirm this observation, we measured *corA* transcripts using RT-qPCR in both CorA^+^ KD100 and CorA^−^ KD101. The transposon insertion site in *corA* locus in KD101 is approximately mid-sequence of the coding region of *corA*, resulting in normal transcription of the first half of the *corA* gene and predictably producing a chimeric transcript between the first half of *corA* mRNA sequence before the junction with the *lacZ* gene of the transposon. Primers for *corA* were, thus, designed in the coding region before the transposon insertion site. As seen in Table 2, the level of *corA* transcript in the *CorA^−^* KD101 is approximately three-fold higher than the level in CorA^+^ KD100 using *recA* gene as the standard. To evaluate whether the mere presence of CorA or its transport function influenced the expression of *corA*, we measured the expression of *corA* in a CorA+ strain in which the transport function was inhibited by growth in the presence of CorA transport inhibitor, Co (III)Hex. Growth in the presence of the CorA transport inhibitor causes the expression of *corA* gene to mimic that in a *corA* mutant (Figure 4). In other words, even though CorA is physically present, the inhibition of its transport function leads to the altered expression of *corA* in a manner similar to the expression in *corA* mutant. This suggest that *corA* is governed by autoregulation, specifically autorepression, which is linked to its transport function.

### 3.5. corA Expression Is HrpL-Dependent

**Figure 5:**
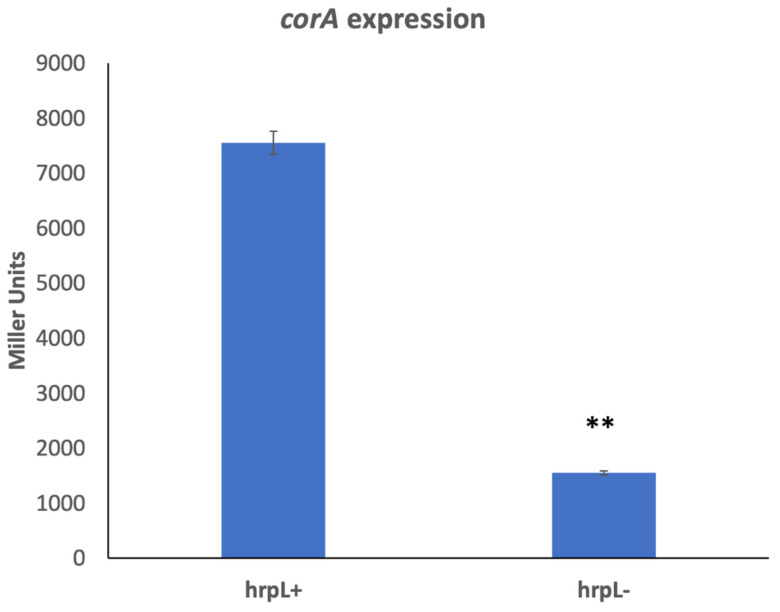
*corA* expression in the absence of HrpL. *P. versatile* KD100 (HrpL^+^) and AC5086 (HrpL^−^) carrying pCKD120 (cosmid-borne *corA-lacZ)* were grown in MM supplemented with tetracycline (10 mg/L). The experiment was repeated twice, and each treatment has three replicates. ** indicates a significant difference where *p* < 0.001 as determined by a Student’s *t*-test.

To further explore the expression *of corA*, we investigated the possibility that *corA* expression is influenced by one of the many regulators known to control exoenzyme production and virulence in *P. versatile*. To determine if this is the case, we measured the expression of cosmid-borne *corA-lacZ* (pCKD120) in a series of exoenzyme and virulence regulatory mutants of *P. versatile* (our lab collection). These regulatory mutants included strains AC5091 (*ahlI^−^*), AC5070 (*rsmA*^−^), AC5050 (*rsmC^−^*), AC5057 (*gacA^−^*), and AC5086 (*hrpL^−^*). The expression levels of *corA-lacZ* in these mutants range from 100 to 70% of wild type levels in *ahlI^−^*, *rsmA*^−^, *rsmC^−^*, and *gacA^−^* strains (data not shown) to a surprising and highly significant decrease of 80% in a *hrpL^−^* background (Figure 5). To confirm this, *corA* transcript levels were measured by RT-qPCR in HrpL^+^ and HrpL^−^ strains. Compared to the HrpL^+^ parent, there is an approximately four-fold decrease in the transcript levels of *corA* in the HrpL^−^ strain compared to the levels in HrpL^+^ strain (Table 2), further supporting the idea that *corA* is regulated by *hrpL*. The expression of type III secretion system effector gene *hrpN* (a known HrpL-regulated gene) was measured by RT-qPCR as a control in the HrpL^−^ strain and was predictably highly reduced (Table 2).

**Figure 6:**
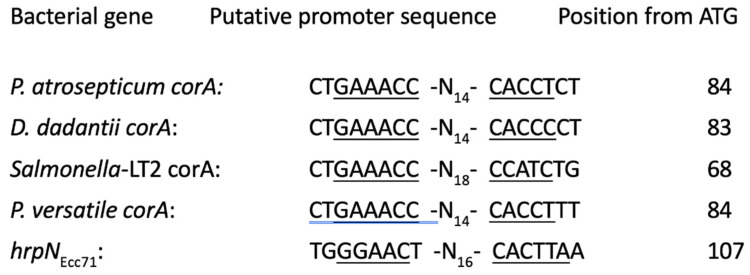
Alignment of possible Hrp box of *corA* in *Pectobacterium*, *Dickeya*, and *Salmonella* species. The underlined nucleotides of the −35 and −10 region represent the putative HrpL promoter sequences. The sequences are aligned with the *hrp* box of *hrpN_Ecc71_*, a confirmed HrpL-regulated gene in *Pectobacterium versatile*.

HrpL is a sigma factor that activates the promoters of many genes involved in type III secretion system and pathogenicity [11]. Paramount among these are the hypersensitive response and pathogenicity (*hrp*) genes, which code for components of type III secretion system as well as harpins and other effectors and avirulence factors [14,45]. The recognition sequences of HrpL sigma factor, termed Hrp box, have been defined at the promoters of its target genes. HrpL recognizes and binds to the consensus sequence GGCCAA-N_16_-CCACNNA, but variations of the sequence have also been reported [46,47]. As our data demonstrate HrpL regulation of *corA* expression, we wanted to find out if the regulatory sequences of *corA* contain a putative *hrpL* recognition sequence. The upstream sequences of *corA* from the soft rot Pectobacteriaceae and *S. enterica* serovar Typhimurium were aligned to search for *hrpL* promoter-like sequences. The sequence GAAACC -N14-CACCT exists in *P. versatile* Ecc71, *P. atrosepticum* SCRI1043, and *Dickeya dadantii* 3937 and GAAACC-N_19_-CCANC in *S. enterica* serovar Typhimurium LT2 and are found approximately 84 and 68 bases away from their respective translational start sites (Figure 6). There is only a slight variation in the putative *hrpL* promoter sequences in the soft rot Pectobacteriaceae shown in Figure 6 from the previously reported [48] Hrp box promoter sequence found in *hrpN_Ecc_* with a difference of one base variation (GAAACC instead of GGAACC) in the −35 site and (CACCT instead of CACTT) in the −10 site. The presence of a similar HrpL-regulated promoter sequence flanking *corA* gives further support that *corA* could be a component of HrpL regulon in *P*. *versatile.*

## 4. Discussion

A *corA* mutation in *P. versatile* led to decreased exoenzyme production and reduced virulence [24]. Although CorA is a major Mg^2+^ transporter, we observed no reduction in intracellular Mg^2+^ in the CorA^−^ mutant compared to the parent. Our observation supports the previous report of a CorA^−^ mutant of *S*. *enterica* serovar Typhimurium [49], in which the intracellular Mg^2+^ content did not appear to be linked to the altered virulence in a CorA^−^ mutant. We did not detect intracellular Ni^2+^ or Co^2+^ in either parent or the mutants. Both metals are also substrates for CorA, and any of them could play a role in the CorA^−^ mutant phenotype. However, both metals have alternative transporters, MgtE for Co^2+^ and a putative high-affinity Ni^2+^ transporter of the NicO superfamily (domain cl00964, PC1_3041) present in *P. versatile* for nickel. Therefore, it is unlikely that a deficiency in the intracellular content of either is responsible for the mutant phenotype. Additionally, cobalt at certain levels is toxic for bacterial growth, although this does not rule out the possibility of its requirement for growth and virulence at sub-inhibitory concentrations.

We investigated if the transport function of CorA is connected to exoenzyme production. We created a condition of physically present and functionally (in terms of transport) deficient CorA by growing CorA^+^ strain in the presence of the CorA inhibitor Co (III) Hex. It has previously been established [34] that Co(III)Hex blocks the transport of magnesium, cobalt, and nickel in *Salmonella enterica*. Co(III)Hex interferes with normal cobalt transport (Figure 1C), as a CorA^+^ strain normally unable to grow in the presence of a high concentration of cobalt is uninhibited in growth. Inhibition of CorA transport function did not produce the CorA^−^ phenotype of lower exoenzymes, suggesting that the physical presence of CorA might be important for CorA’s role in exoenzyme production and virulence. This observation is consistent with our earlier report that *corA* strains are severely affected in multiplication and survival in the host tissues [24]. Thus, we postulate that CorA’s role in virulence of *Pectobacterium* could lie solely in its physical presence in two ways. The first of these is reduced PCWDEs production and the second is multiplication and survival in the host tissues. A similar observation and suggestion have also been made for CorA in *S. enterica* serovar Typhimurium [30]. While we clarified that the impact of CorA on exoenzyme production is, at least in part, at the transcriptional level, we are yet to determine its regulatory mechanism. We know of no other such system besides CorA in *Salmonella* and *Pectobacterium*.

As measured by the expression of *corA–lacZ* fusion and RT-qPCR, CorA auto-represses its own expression in *P. versatile*, as *corA* expression is increased in the absence of CorA. Interestingly, in the presence of 250 µM CorA inhibitor, the autorepression is cancelled and the expression of *corA* in a CorA^+^ strain is the same as its expression in a CorA^−^ strain, suggesting that CorA transport function may be what is responsible for regulating its own expression. It is not clear to us yet how this autorepression works and what is its significance.

Our data also indicate that the expression of *corA* in *P. versatile* might be regulated by the sigma factor HrpL. A closer look at the promoter region *corA* reveal a putative HrpL recognition sequence, *hrp* box, that differs in only one nucleotide in both the −35 and −10 motif. The *hrp* box is a bipartite nucleotides -GGAACCNA-N_13-14_-CCACNNA at the −35 and −10 sites, respectively [50,51,52]. Mutational analysis of these sequences in *Pantoea agglomeran* pv. *gypsophilae* determines that GGAAC and ACNNA are both essential at the −35 and −10 regions, respectively, for promoter activity through HrpL [53]. The putative *hrp* box of *corA_Ecc71_* is −35 GAAAC and −10 ACNNC. The promoter specificity of the Hrp box is determined by the interaction of region 4 of HrpL and the −35 motif [54]. There is a 15% difference between the region 4 of HrpL proteins of *Pantoea agglomeran* pv. *gypsophilae* and *P. versatile* Ecc71 [12] and this could possibly allow for differences in target promoter specificity between the two organisms. Other HrpL-regulated genes have been reported with similar variations [55,56].

HrpL is a member of the extracytoplasmic function (ECF) subfamily of σ^70^ sigma factors [11,46] which are involved in the transcription of many genes in response to environmental stimuli [57]. As a result, the ECF sigma factors in general are often involved in regulating outer membrane proteins synthesis [58,59]. As CorA is a transmembrane protein involved in Mg^2+^, Co^2+^, and Ni^2+^ transport, its regulation by HrpL would fall in line with the pattern of ECF sigma factor regulation. Metal transporters in other bacteria have also been reported to be regulated by ECF sigma factors. In *Ralstonia metallidurans* (formerly *Alcaligenes eutrophus*) an ECF sigma factor, CrnH, regulates the Co^2+^/Ni^2+^ antiporter CrnA [60]. Additionally, in a HrpL mutant of the related soft rot pathogen *D*. *dadantii*, microarray and bioinformatic analysis revealed differential expression of CorC, a Mg^2+^ and Co^2+^ transporter [61]. Interestingly, *corA* expression was not reported as being under the influence of HrpL, although the *hrp* boxes of *D*. *dadanti* and *P. versatile* differ by only a single nucleotide in the −10 region. That said, it does not necessarily follow that these observations in different organisms translate to similar effects in Ecc71 or that our observations in this study equally apply to other organisms or even other strains of *P. versatile*. It remains unclear what link, if any, exists between CorA’s effect on virulence and its regulation by HrpL in *P*. *versatile* strain Ecc71, the subject of our study. Further investigations are needed to confirm the lines of evidence we have provided here suggesting that CorA is in the HrpL regulon.

## Figures and Tables

**Table 1:** Strains and plasmids.

Bacteria and Plasmids	Relevant Characteristics	Reference or Source
Bacteria		
***Escherichia coli***		
S17-1 λ pir	*recA pro hsdR RP4-2-Tc::Mu-Km::Tn7*	[32]
XL 10 Gold	Δ(mcrA)183 Δ (mcrCB-hsdSMR-mrr)173 endA1 supE44 thi-1 recA1 gyrA96 relA1 lac Hte [F’proAB lacI ^q^ ZΔM15 Tn10(Tet^r^) Amy Cm^r^	Agilent
***Pectobacterium versatile***		
Ecc71	Wild-type	[33]
KD100	*lacZ^−^*, Nal^R^, Derived from Ecc71	[24]
KD101	*corA::lacZ*, Km^R^ of KD100.	[24]
KD103	*corA-EZTN*, Km^R^ of KD100	This study
AC5050	*rsmC* ^−^	[34]
AC5057	*gacA* ^−^	[35]
AC5070	*rsmA* ^−^	[36]
AC5077	*hexA* ^−^	[37]
AC5086	*hrpL* ^−^	[11]
AC5091	*ahlI* ^−^	[36]
**Plasmids**		
pTH19cr	Cm^r^, low copy cloning vector, pSC101 replicon.	[38]
pRK415	Tc^r^, low copy cloning vector	[39]
pMP220	Tc^r^, promoter–probe vector	[40]
pLAFR5	Tc^r^, cosmid cloning vector	[39]
pCKD120	Km^r^, Tc^r^ *corA*^−^::Tn5 *lacZ1* in pLAFR5	[24]
pCKD121	Cm^r^, *corA^+^* DNA in pTH19cr	[24]
pCKD122	*corA^+^* DNA in pRK415, Tc^R^	This study
pCKD123	EZ-TN cassette inserted into *corA* in pCKD122; used to construct KD103.	This study
pAKC1203	Tc^r,^ *pel-1*-*lacZ* in pMP220	[41]

**Table 2:** Comparison of transcript levels in *corA* and *hrpL* mutants of *P. versatile* compared to their parents.

Gene	Relevant Host Genotype	Fold Change §
*corA*	*corA^−^*	+3.11 ± 0.74
*corA*	*hrpL^−^*	−3.95 ± 0.77
*hrpN*	*hrpL^−^*	−17.25 ± 4.74

**§** The fold change in *corA* and *hrpL* mutants compared to their parents was determined by RT-qPCR. Data represent the mean ± SD of 2 individual biological replications.

## Data Availability

All the data relevant to this study are provided in tables and figures. Further information can be requested from the corresponding author.

## References

[1] Charkowski A.O. (2018). The changing face of bacterial soft-rot diseases. Annu. Rev. Phytopathol..

[2] Perombelon M.C.M., Kelman A. (1980). Ecology of the soft rot erwinas. Annu. Rev. Phytopathol..

[3] Barras F., Van Gijsegem F., Chatterjee A.K. (1994). Extracellular enzymes and pathogenesis of soft-rot *Erwinia*. Annu. Rev. Phytopathol..

[4] Hauben L., Moore E.R., Vauterin L., Steenackers M., Mergaert J., Verdonck L., Swings J. (1998). Phylogenetic position of phytopathogens within the Enterobacteriaceae. Syst. Appl. Microbiol..

[5] Portier P., Pédron J., Taghouti G., Fischer-Le Saux M., Caullireau E., Bertrand C., Laurent A., Chawki K., Oulgazi S., Moumni M. (2019). Elevation of *Pectobacterium carotovorum* subsp. odoriferum to species level as *Pectobacterium odoriferum* sp. nov., proposal of *Pectobacterium brasiliense* sp. nov. and *Pectobacterium actinidiae* sp. nov., emended description of *Pectobacterium carotovorum* and description of *Pectobacterium versatile* sp. nov., isolated from streams and symptoms on diverse plants. Int. J. Syst. Evol. Microbiol..

[6] Davidsson P.R., Kariola T., Niemi O., Palva E.T. (2013). Pathogenicity of and plant immunity to soft rot pectobacteria. Front. Plant Sci..

[7] Ma B., Hibbing M.E., Kim H.S., Reedy R.M., Yedidia I., Breuer J., Glasner J.D., Perna N.T., Kelman A., Charkowski A.O. (2007). Host range and molecular phylogenies of the soft rot enterobacterial genera *Pectobacterium* and *Dickeya*. Phytopathology.

[8] Joshi J.R., Khazanov N., Charkowski A., Faigenboim A., Senderowitz H., Yedidia I. (2021). Interkingdom Signaling interference: The effect of plant-derived small molecules on quorum sensing in plant-pathogenic bacteria. Annu. Rev. Phytopathol..

[9] Chatterjee A.K., Dumenyo C.K., Liu Y., Chatterjee A., Lederberg J. (2000). *Erwinia*: Genetics of pathogenicity factors. Encyclopedia of Microbiology.

[10] Thomson N.R., Thomas J.D., Salmond G.P.C., Margaret C.M.S., Sockett R.E. (1999). Virulence determinants in the bacterial phytopathogen *Erwinia*. Methods Microbiol.

[11] Chatterjee A., Cui Y., Chatterjee A.K. (2002). Regulation of *Erwinia carotovora hrpL_Ecc_* (Sigma-L_Ecc_), which encodes an extracytoplasmic function subfamily of sigma factor required for expression of the HRP regulon. Mol. Plant Microbe Interact..

[12] Chatterjee A., Cui Y., Chaudhuri S., Chatterjee A.K. (2002). Identification of regulators of *hrp/hop* genes of *Erwinia carotovora* ssp. *carotovora* and characterization of HrpL_Ecc_ (SigmaL_Ecc_), an alternative sigma factor. Mol. Plant Pathol..

[13] Salmond G.P.C. (1994). Secretion of Extracellular Virulence Factors by Plant Pathogenic Bacteria. Annu. Rev. Phytopathol..

[14] Rantakari A., Virtaharju O., Vahamiko S., Taira S., Palva E.T., Saarilahti H.T., Romantschuk M. (2001). Type III secretion contributes to the pathogenesis of the soft-rot pathogen *Erwinia carotovora*: Partial characterization of the *hrp* gene cluster. Mol. Plant. Microbe Interact..

[15] Hogan C.S., Mole B.M., Grant S.R., Willis D.K., Charkowski A.O. (2013). The type III secreted effector DspE is required early in solanum tuberosum leaf infection by *Pectobacterium carotovorum* to cause cell death, and requires Wx(3-6)D/E motifs. PLoS ONE.

[16] Condemine G., Robert-Baudouy J. (1987). 2-keto-3-deoxygluconate transport system in *Erwinia chrysanthemi*. J. Bacteriol..

[17] Haseloff B.J., Freeman T.L., Valmeekam V., Melkus M.W., Oner F., Valachovic M.S., San Francisco M.J. (1998). The *exuT* gene of *Erwinia chrysanthemi* EC16: Nucleotide sequence, expression, localization, and relevance of the gene product. Mol. Plant Microbe Interact..

[18] Hugouvieux-Cotte-Pattat N., Reverchon S. (2001). Two transporters, TogT and TogMNAB, are responsible for oligogalacturonide uptake in *Erwinia chrysanthemi* 3937. Mol. Microbiol..

[19] Hugouvieux-Cotte-Pattat N., Blot N., Reverchon S. (2001). Identification of TogMNAB, an ABC transporter which mediates the uptake of pectic oligomers in *Erwinia chrysanthemi* 3937. Mol. Microbiol..

[20] Urbany C., Neuhaus H.E. (2008). Citrate uptake into *Pectobacterium atrosepticum* is critical for bacterial virulence. Mol. Plant. Microbe Interact..

[21] Barabote R.D., Johnson O.L., Zetina E., San Francisco S.K., Fralick J.A., San Francisco M.J. (2003). *Erwinia chrysanthemi* tolC is involved in resistance to antimicrobial plant chemicals and is essential for phytopathogenesis. J. Bacteriol..

[22] Maggiorani Valecillos A., Rodríguez Palenzuela P., López-Solanilla E. (2006). The role of several multidrug resistance systems in *Erwinia chrysanthemi* pathogenesis. Mol. Plant Microbe Interact..

[23] Gloux K., Touze T., Pagot Y., Jouan B., Blanco C. (2005). Mutations of *ousA* alter the virulence of *Erwinia chrysanthemi*. Mol. Plant Microbe Interact..

[24] Kersey C.M., Agyemang P.A., Dumenyo C.K. (2012). CorA, the magnesium/nickel/cobalt transporter, affects virulence and extracellular enzyme production in the soft rot pathogen *Pectobacterium carotovorum*. Mol. Plant Pathol..

[25] Romani A.M., Scarpa A. (2000). Regulation of cellular magnesium. Front. Biosci.-Landmark.

[26] Yin X., Wu Orr M., Wang H., Hobbs E.C., Shabalina S.A., Storz G. (2019). The small protein MgtS and small RNA MgrR modulate the PitA phosphate symporter to boost intracellular magnesium levels. Mol. Microbiol..

[27] Maguire M.E. (2006). Magnesium transporters: Properties, regulation and structure. Front. Biosci.-Landmark.

[28] Wang H., Yin X., Wu Orr M., Dambach M., Curtis R., Storz G. (2017). Increasing intracellular magnesium levels with the 31-amino acid MgtS protein. Proc. Natl. Acad. Sci. USA.

[29] Smith R.L., Maguire M.E. (1995). Distribution of the CorA Mg^2+^ transport system in gram-negative bacteria. J. Bacteriol..

[30] Papp-Wallace K.M., Nartea M., Kehres D.G., Porwollik S., McClelland M., Libby S.J., Fang F.C., Maguire M.E. (2008). The CorA Mg^2+^ channel is required for the virulence of Salmonella enterica serovar typhimurium. J. Bacteriol..

[31] Sambrook J., Russell D.W. (2006). The Condensed Protocols from Molecular Cloning: A Laboratory Manual.

[32] Simon R., Priefer U., Puhler A. (1983). A broad host range mobilization system for in vivo genetic-engineering: Transposon mutagenesis in gram-negative bacteria. Biotechnology.

[33] Zink R.T., Engwall J.K., McEvoy J.L., Chatterjee A.K. (1985). recA is required in the induction of pectin lyase and carotovoricin in *Erwinia carotovora* subsp. carotovora. J. Bacteriol..

[34] Cui Y., Mukherjee A., Dumenyo C.K., Liu Y., Chatterjee A.K. (1999). rsmC of the soft-rotting bacterium *Erwinia carotovora* subsp. carotovora negatively controls extracellular enzyme and harpin(Ecc) production and virulence by modulating levels of regulatory RNA (rsmB) and RNA-binding protein (RsmA). J. Bacteriol..

[35] Cui Y., Chatterjee A., Yang H., Chatterjee A.K. (2008). Regulatory network controlling extracellular proteins in *Erwinia carotovora* subsp. carotovora: FlhDC, the master regulator of flagellar genes, activates rsmB regulatory RNA production by affecting gacA and hexA (lrhA) expression. J. Bacteriol..

[36] Chatterjee A., Cui Y., Liu Y., Dumenyo C.K., Chatterjee A.K. (1995). Inactivation of rsmA leads to overproduction of extracellular pectinases, cellulases, and proteases in *Erwinia carotovora* subsp. carotovora in the absence of the starvation/cell density-sensing signal, N-(3-oxohexanoyl)-L-homoserine lactone. Appl. Environ. Microbiol..

[37] Mukherjee A., Cui Y., Ma W., Liu Y., Chatterjee A.K. (2000). hexA of *Erwinia carotovora* ssp. carotovora strain Ecc71 negatively regulates production of RpoS and rsmB RNA, a global regulator of extracellular proteins, plant virulence and the quorum-sensing signal, N-(3-oxohexanoyl)-L-homoserine lactone. Environ. Microbiol..

[38] Hashimoto-Gotoh T., Yamaguchi M., Yasojima K., Tsujimura A., Wakabayashi Y., Watanabe Y. (2000). A set of temperature sensitive-replication/-segregation and temperature resistant plasmid vectors with different copy numbers and in an isogenic background (chloramphenicol, kanamycin, lacZ, repA, par, polA). Gene.

[39] Keen N.T., Tamaki S., Kobayashi D., Trollinger D. (1988). Improved broad-host-range plasmids for DNA cloning in Gram-negative bacteria. Gene.

[40] Spaink H.P., Okker R.J.H., Wijffelman C.A., Pees E., Lugtenberg B.J.J. (1987). Promoters in the nodulation region of the *Rhizobium leguminosarum* Sym plasmid pRL1JI. Plant Mol. Biol..

[41] Cui Y., Chatterjee A., Hasegawa H., Dixit V., Leigh N., Chatterjee A.K. (2005). ExpR, a LuxR homolog of *Erwinia carotovora* subsp. carotovora, activates transcription of rsmA, which specifies a global regulatory RNA-binding protein. J. Bacteriol..

[42] Miller J.H. (1972). Experiments in Molecular Genetics.

[43] Liu Y., Chatterjee A., Chatterjee A.K. (1994). Nucleotide sequence and expression of a novel pectate lyase gene (pel-3) and a closely linked endopolygalacturonase gene (peh-1) of *Erwinia carotovora* subsp. carotovora 71. Appl. Environ. Microbiol..

[44] Kucharski L.M., Lubbe W.J., Maguire M.E. (2000). Cation hexaammines are selective and potent inhibitors of the CorA magnesium transport system. J. Biol. Chem..

[45] Lehtimaki S., Rantakari A., Routtu J., Tuikkala A., Li J., Virtaharju O., Palva E.T., Romantschuk M., Saarilahti H.T. (2003). Characterization of the hrp pathogenicity cluster of *Erwinia carotovora* subsp. carotovora: High basal level expression in a mutant is associated with reduced virulence. Mol. Genet. Genom..

[46] Wei Z.M., Beer S.V. (1995). hrpL activates *Erwinia amylovora* hrp gene transcription and is a member of the ECF subfamily of sigma factors. J. Bacteriol..

[47] Vencato M., Tian F., Alfano J.R., Buell C.R., Cartinhour S., DeClerck G.A., Guttman D.S., Stavrinides J., Joardar V., Lindeberg M. (2006). Bioinformatics-enabled identification of the HrpL regulon and type III secretion system effector proteins of *Pseudomonas syringae* pv. phaseolicola 1448A. Mol. Plant Microbe Interact..

[48] Mukherjee A., Cui Y., Liu Y., Chatterjee A.K. (1997). Molecular characterization and expression of the *Erwinia carotovora* hrpNEcc gene, which encodes an elicitor of the hypersensitive reaction. Mol. Plant Microbe Interact..

[49] Papp-Wallace K.M., Maguire M.E. (2008). Regulation of CorA Mg2+ channel function affects the virulence of *Salmonella enterica* serovar Typhimurium. J. Bacteriol..

[50] Xiao Y., Heu S., Yi J., Lu Y., Hutcheson S.W. (1994). Identification of a putative alternate sigma factor and characterization of a multicomponent regulatory cascade controlling the expression of *Pseudomonas syringae* pv. syringae Pss61 hrp and hrmA genes. J. Bacteriol..

[51] Shen H., Keen N.T. (1993). Characterization of the promoter of avirulence gene D from *Pseudomonas syringae* pv. tomato. J. Bacteriol..

[52] Innes R.W., Bent A.F., Kunkel B.N., Bisgrove S.R., Staskawicz B.J. (1993). Molecular analysis of avirulence gene avrRpt2 and identification of a putative regulatory sequence common to all known *Pseudomonas syringae* avirulence genes. J. Bacteriol..

[53] Nissan G., Manulis S., Weinthal D.M., Sessa G., Barash I. (2005). Analysis of promoters recognized by HrpL, an alternative sigma-factor protein from *Pantoea agglomerans* pv. gypsophilae. Mol. Plant Microbe Interact..

[54] Lonetto M., Gribskov M., Gross C.A. (1992). The sigma 70 family: Sequence conservation and evolutionary relationships. J. Bacteriol..

[55] Fouts D.E., Abramovitch R.B., Alfano J.R., Baldo A.M., Buell C.R., Cartinhour S., Chatterjee A.K., D’Ascenzo M., Gwinn M.L., Lazarowitz S.G. (2002). Genomewide identification of *Pseudomonas syringae* pv. tomato DC3000 promoters controlled by the HrpL alternative sigma factor. Proc. Natl. Acad. Sci. USA.

[56] Thwaites R., Spanu P.D., Panopoulos N.J., Stevens C., Mansfield J.W. (2004). Transcriptional regulation of components of the type III secretion system and effectors in *Pseudomonas syringae* pv. phaseolicola. Mol. Plant Microbe Interact..

[57] Helmann J.D. (2002). The extracytoplasmic function (ECF) sigma factors. Adv. Microb. Physiol..

[58] Kazmierczak M.J., Wiedmann M., Boor K.J. (2005). Alternative sigma factors and their roles in bacterial virulence. Microbiol. Mol. Biol. Rev..

[59] Brooks B.E., Buchanan S.K. (2008). Signaling mechanisms for activation of extracytoplasmic function (ECF) sigma factors. Biochim. Biophys. Acta.

[60] Taghavi S., Mergeay M., Nies D., van der Lelie D. (1997). *Alcaligenes eutrophus* as a model system for bacterial interactions with heavy metals in the environment. Res. Microbiol..

[61] Yang S.H., Peng Q.A., Zhang Q., Zou L.F., Li Y., Robert C., Pritchard L., Liu H., Hovey R., Wang Q. (2010). Genome-Wide Identification of HrpL-Regulated Genes in the Necrotrophic Phytopathogen *Dickeya dadantii* 3937. PLoS ONE.

